# *Micromonosporaceae* biosynthetic gene cluster diversity highlights the need for broad-spectrum investigations

**DOI:** 10.1099/mgen.0.001167

**Published:** 2024-01-04

**Authors:** Imraan Alas, Doug R. Braun, Spencer S. Ericksen, Rauf Salamzade, Lindsay Kalan, Scott R. Rajski, Tim S. Bugni

**Affiliations:** ^1^​ Pharmaceutical Sciences Division, University of Wisconsin–Madison, Madison, WI, USA; ^2^​ Small Molecule Screening Facility, UW Carbone Cancer Center, Madison, WI, USA; ^3^​ Department of Medical Microbiology & Immunology, University of Wisconsin–Madison, Madison, WI, USA; ^4^​ Department of Biochemistry & Biomedical Sciences, McMaster University, Health Sciences Centre, Hamilton, ON, Canada; ^5^​ Lachman Institute for Pharmaceutical Development, University of Wisconsin–Madison, Madison, WI, USA

**Keywords:** biosynthetic gene cluster, drug discovery, genomics, marine-derived, natural products

## Abstract

Investigations of the bacterial family *Micromonosporaceae* have enabled the development of secondary metabolites critical to human health. Historical investigation of bacterial families for natural product discovery has focused on terrestrial strains, where time-consuming isolation processes often lead to the rediscovery of known compounds. To investigate the secondary metabolite potential of marine-derived *Micromonosporaceae*, 38 strains were sequenced, assembled and analysed using antiSMASH and BiG-SLiCE. BiG-SLiCE contains a near-comprehensive dataset of approximately 1.2 million publicly available biosynthetic gene clusters from primarily terrestrial strains. Our marine-derived *Micromonosporaceae* were directly compared to BiG-SLiCE’s preprocessed database using BiG-SLiCE’s query mode; genetic diversity within our strains was uncovered using BiG-SCAPE and metric multidimensional scaling analysis. Our analysis of marine-derived *Micromonosporaceae* emphasizes the clear need for broader genomic investigations of marine strains to fully realize their potential as sources of new natural products.

## Abbreviations

AAI, average amino acid identity; ANI, average nucleotide identity; BGC, biosynthetic gene cluster; GCF, gene cluster family; MDR, multi-drug-resistant; MDS, multidimensional scaling; NP, natural product; NRPS, non-ribosomal peptide synthetase; PKS, polyketide synthase; RiPPs, ribosomally synthesized and post-translationally modified peptides; SSN, sequence similarity network.

## Impact Statement

The increasingly deadly threat to humanity posed by drug-resistant and completely new infectious diseases continues to drive efforts to expedite and expand efforts to identify new antimicrobial agents as therapeutics and drug leads. Microbial producers of, as yet, undiscovered new small molecules with medicinal potential have originated historically from terrestrial sources. However, it is becoming increasingly apparent that marine environments harbour repositories of microbial small molecule producers that rival, if not surpass, the structural and biosynthetic diversities of terrestrial organisms. Our work here supports this notion, and given recent advances in bioinformatics, culturing methodologies and available analytical instrumentations, seeks to illuminate the potential of marine-derived microbes as beacons for discovery of new anti-infective drug leads. Careful comparisons of marine genomics information to databases obtained from terrestrial microbes bears out the clear need to better exploit marine-derived microbes as points of discovery and inspiration. This work seeks to impact the field of microbial genomics and drug discovery by showcasing marine-derived microbes as high-priority opportunities for the discovery of new and probably novel biosynthetic machineries and their resulting natural products.

## Data Summary

Large datasets (mostly as tables) and special files can be found in Zenodo (https://zenodo.org/) and Figshare (https://figshare.com/) under the following links: https://zenodo.org/record/8137859 and https://doi.org/10.6084/m9.figshare.24492253.v1. In particular, the following collections of data for this paper are included:

- **Data S1**: A folder with all the fasta files, representing the 42 strains (41 *Micromonosporaceae*, 1 *Streptomycetaceae*).
- **Data S2**: A folder with all the .gbk files for the biosynthetic gene cluster (BGC) regions predicted by antiSMASH v5.1.1. These files were used as inputs for BiG-SCAPE and BiG-SLiCE.
- **Data S3**: A folder with all the .gbk files for the BGC regions predicted by antiSMASH v6.1.0.
- **Data S4**: A folder containing all the Quast outputs for the 42 strains.
- **Data S5**: A folder containing all the BUSCO outputs for the 42 strains. Example scripts are provided for scraping relevant information from the individual BUSCO outputs.
- **Data S6**: A folder containing GTDB (Genome Taxonomy Database) classification results, and species-level grouping results using FastANI (95 % cutoff).
- **Data S7**: A folder containing an Interactive Tree of Life (iTOL)-compatible bar chart annotation using antiSMASH v5.1.1 BGC region information.
- **Data S8**: A folder containing a Word document that describes the parameters used with Ubuntu WSL (Windows Subsystem for Linux) on the command line for programs antiSMASH v6.1.0, BiG-SCAPE v1.1.2 and BiG-SLiCE v1.1.1. Also included are parameters for metric MDS in python. An example script is also provided for batch queries of BGCs against BiG-SLiCE v1.1.1’s pre-processed dataset of ~1.2 million BGCs.
- **Data S9**: A folder containing the BiG-SCAPE visualization of the 38 *Micromonosporaceae* (post-QC filtering, excluding WMMA1363, WMMB482, WMMB486 and WMMC500) in Cytoscape.
- **Data S10**: A folder containing:
  - The pre-processed dataset of 1.2 million BGCs from BiG-SLiCE.
  - All report folders generated by BiG-SLiCE for the 779 *Micromonosporaceae* BGCs queried against the 1.2 million BGCs.
  - The results data.db and associated folders for the pre-processed dataset of 1.2 million BGCs.
- **Data S11**: A folder containing the scripts necessary to regenerate the figures and perform independent analyses, and the relevant data used for the analyses.
- **Data S12:** A folder containing the NCBI blast query used to compare WMMD1947 region 12 against WMMD1120 region 14 using antiSMASH v6.1.0.
- **Data S13:** A folder containing the files pertaining to RiPP subclasses, BiG-SLiCE BGCs annotated with BiG-SCAPE information, and MIBiG BGCs identified as being related to BGCs in our dataset.

In addition to having provided these materials through Zenodo and FigShare, the authors confirm that all supporting data, code and protocols have been provided within the article or through supplementary data files.

## Introduction

Drug-resistant infectious diseases have been recognized for decades as a growing threat to humanity [1, 2]. More recent crises such as sudden respiratory syndrome (SARS), monkey pox and, most dramatically, the coronavirus disease 2019 (COVID-19) pandemic have exacerbated the issue significantly due to the increased use of antimicrobials and the predictable acceleration of healthcare-associated drug-resistant infections in US hospitals [3, 4]. In tandem with these realizations, it has also long been recognized that bacterially derived secondary metabolites (natural products, NPs) constitute an idealized repository of new drug leads with the potential to display novel mechanisms of action, and thus the ability to circumvent current drug resistance mechanisms in pathogens [2, 5, 6]. However, decades of mining terrestrial sources for useful NPs have reduced the likelihood of identifying truly new and novel NPs, despite truly remarkable technical advances that have streamlined drug discovery processes [5, 7]. In contrast, recent efforts have revealed marine-derived microbes to be extremely attractive and, now, tractable sources; this is especially true for actinobacterial populations [7–13]. For instance, the marine actinomycete genus *Salinispora* was prioritized for novel NP explorations and subsequently enabled the discovery of lomaiviticins, salinosporamides and other antibacterial compounds [13].

Despite their inability to grow in deionized water compared to seawater, *Salinispora* have been disproportionately studied within the marine-derived NP community; the breadth of *Salinispora* studies to date has no doubt been a result of their well-noted and chronologically early recognition as sources of NP originality [13–16]. Perceived limitations of *Salinispora*, as a genus, and technological advances of the last 10 years, have inspired recent campaigns to aggressively evaluate other marine-derived bacteria as sources for new NP scaffolds [12].

For example, phylogenetic analyses of brackish water sponges unveiled proportionally more *Micromonospora* spp. compared to *Streptomyces* spp., contrasting earlier analyses of soil environment isolates [9, 17]. Moreover, comparisons of brackish water habitats and tropical reef-type habitats have revealed an abundance of four specific genera: *Streptomyces* spp., *Micromonospora* spp., *Solwaraspora* spp. and *Verrucosispora* spp. [9]. With distributions of bacterial taxa differing across environments, notable metabolic diversity within *Micromonospora* spp., and the historical emphasis on *Streptomyces* spp. exploration, it is now clear that investigations of marine *Micromonosporaceae* are likely to unveil NPs that differ from those of terrestrial strains, including both general terrestrial bacterial strains and terrestrial *Micromonosporaceae Micromonospora* spp. [9, 17, 18]. (This study showed that members of the family *Micromonosporaceae* were more abundant among cultivated bacteria from tropical ecosystems versus brackish ones. In addition, metabolomics initiatives have shown their metabolites to differ substantially from those of brackish *Streptomyces.*)

Notably, the potential of *Micromonospora* to generate antimicrobials was first recognized in 1947 with the discovery of the polyketide micromonosporin and subsequently underscored with the discovery of gentamicin (Gentocin, Garamycin) from *Micromonospora purpurea* in 1963; importantly, gentamicin is listed by the World Health Organization (WHO) as an essential and critically important medication [19–21]. In the time intervening gentamicin’s discovery and now, over 740 antibiotics have been discovered from *Micromonospora* strains although remarkably few have received clinical approval. Not surprisingly, the metabolic capacities of *Micromonospora* family members and their impact on drug discovery initiatives have been the subject of several outstanding reviews in recent years [7, 18, 22]. Gentamicin, netilimicin, plazomicin (Zemdri), isepamicin, neomycin (neo-Fradin, neo-Tab) and sisomicin (bactoCeaze, Ensamycin) represent, by far, the most clinically successful *Micromonospora*-derived agents and all remain in service today to differing extents based on geographical location and specific antimicrobial applications [18, 22]. In addition to aminoglycosides, *Micromonospora* are known to produce antimicrobials bearing macrolide, ansamycin, everninomicin and actinomycin scaffolds [7, 18, 22, 23]. In addition to serving as sources of antimicrobials with activity against ‘susceptible pathogens’, *Micromonospora* have also yielded metabolites with potent activity against multiple-drug-resistant (MDR) microbes. For instance, turbinmicin, reported in 2020 from *Micromonospora* WMMC-415, displays broad-spectrum activity against MDR fungal pathogens, notably *Candida auris* and *Aspergillus fumigatus* [24, 25] and is currently in clinical development. In addition, *Micromonospora* have been shown to produce the enediyne-based DNA-damaging agents calicheamicin, yangpumicins and dynemicin; all hail from *Micromonospora* and have been applied to the creation and use of human therapeutics to differing extents [7, 18, 22, 26–28]. Thus, it is now abundantly clear that *Micromonospora* and associated family members of the *Micromonosporaceae* represent outstanding resources for NP discovery, and highlight environments that produce these bacteria as primary investigative targets.

Concurrent with shifting views on the importance of marine vs. terrestrial NP repositories has been the continuous advancement of technologies aimed at acquiring and exploiting genetic information, specifically the development of high-throughput DNA sequencing methods. Compared to traditional 16S rRNA gene sequencing and phylogenetic strain classification, whole genome sequencing as used by the Genome Taxonomy Database has allowed for enhanced disambiguation of genomes/organisms [29, 30]. Average nucleotide identity (ANI), accessible via whole genome sequencing, now enables novel bacteria to be better understood relative to publicly available literature; one result of this advance has been the ability to delineate classically understood genera into more representative genera [30]. Additional genomic information acquired via whole genome sequencing has shed significant light on orphan biosynthetic gene clusters (BGCs) housed within bacteria and fungi [31]. BGCs represent groupings of genes involved with the production of secondary metabolites, thus linking genomics to the production and intracellular use of bioactive NPs [32]. Relatedly, the refinement of antiSMASH (antibiotics and Secondary Metabolite Analysis Shell) has enabled the expanded exploitation of bacterial genomes in the search for antimicrobial NPs [33]. In conjunction, the construction of databases from public literature sources such as MIBiG (Minimum Information about a Biosynthetic Gene cluster) has allowed for improved use of already described BGCs in the search for new NPs [32].

Importantly, tools such as antiSMASH and MIBiG can be utilized by downstream software such as BiG-SCAPE (Biosynthetic Gene Similarity Clustering and Prospecting Engine) and BiG-SLiCE (Biosynthetic Gene clusters – Super Linear Clustering Engine) to create sequence similarity networks (SSNs) across strain collections [32–35]. These genome-guided computational tools enable one to identify uncharacterized BGCs and their related products across large collections of strains and employ both publicly available literature and privately held datasets [36]. Such an approach minimizes the likelihood of NP rediscovery, a well-known hurdle to effective drug discovery efforts and maximizes currently known correlations of genomic information to small molecule structure and function. These newfound capabilities are ideally suited to performing critical analyses regarding new NP potentials of any collection of microbial NP producers one might wish to interrogate.

To date, a broad analysis of marine *Micromonosporaceae* genomes, BGCs related to the production of NPs and their relation to public databases has been lacking. Hence, we provide here a comparative genomic analysis of marine *Micromonosporaceae* with the intention of identifying truly novel BGCs. Our analyses revealed the biosynthetic diversity of marine *Micromonosporaceae*, especially in comparison to publicly curated databases. Interestingly, we identified four new species belonging to *Micromonospora_E*, which is a new genus within the GTDB-tk2 database. These species represent a 4-fold increase in known *Micromonosporaceae Micromonospora_E* compared to the 311 480 bacterial strains stored in GTDB-tk2 from publicly available data. Further investigations necessitated the development of a novel methodology to visually explore BGC similarity across an individual strain collection and against publicly available data, to prioritize unique BGCs for identification and investigation . This study showcases: (a) the utility of prioritizing BGCs based on novelty, relative to large existing databases; and (b) the potential of renewed investigations into marine *Micromonosporaceae* as an outstanding repository for NP scaffolds.

## Methods

### Strain isolation and extraction

Bacterial strains were isolated using previously published methodologies [37]. 16S rRNA gene sequences were extracted using standard methods [37]. DNA sequences were extracted using standard protocols for our laboratory [38].

### Strain sequencing, assembly and validation

Next-generation sequencing was performed with PacBio Sequel platforms using two Sequel single-molecule real-time (SMRT) cells [University of Wisconsin, Madison (UW-Madison), Biotechnology Center]. PacBio data were corrected, trimmed and assembled with Canu v1.8 [39] using the parameter ‘genomeSize=8m’ (UW-Madison, Center for High Throughput Computing). BUSCO v5.4.3 [40] and QUAST v5.0.2 [41] were used to assess each genome assembly based on completeness and quality respectively, and the results are listed in Data S4 and S6 (available in the online version of this article).

### Annotation of biosynthetic gene clusters

To identify BGCs related to secondary metabolism, genome sequences in Fasta format were annotated by installations of antiSMASH v5.1.1 [42] and antiSMASH v6.1.0 [43]. For antiSMASH v5.1.1, a minimal run omitting additional features was carried out using Antibiotic Resistant Target Seeker Version 2 (ARTS) [44, 45]. For antiSMASH v6.1.0, BGC prediction was performed with detection strictness relaxed, and extra features KnownClusterBlast, ActiveSiteFinder, SubClusterBlast and RREFinder were active. The full parameters for the antiSMASH runs are listed in Data S8. Detailed information for the 39 strains is listed in Data S4–S6.

A pie plot of overall BGC counts per predicted product type was constructed using seaborn (v0.11.2) [46] in Python (v3.7.12) [47].

### Phylogenomics, average identity estimation between genomic pairs and taxonomic classification

**Fig. 1.**
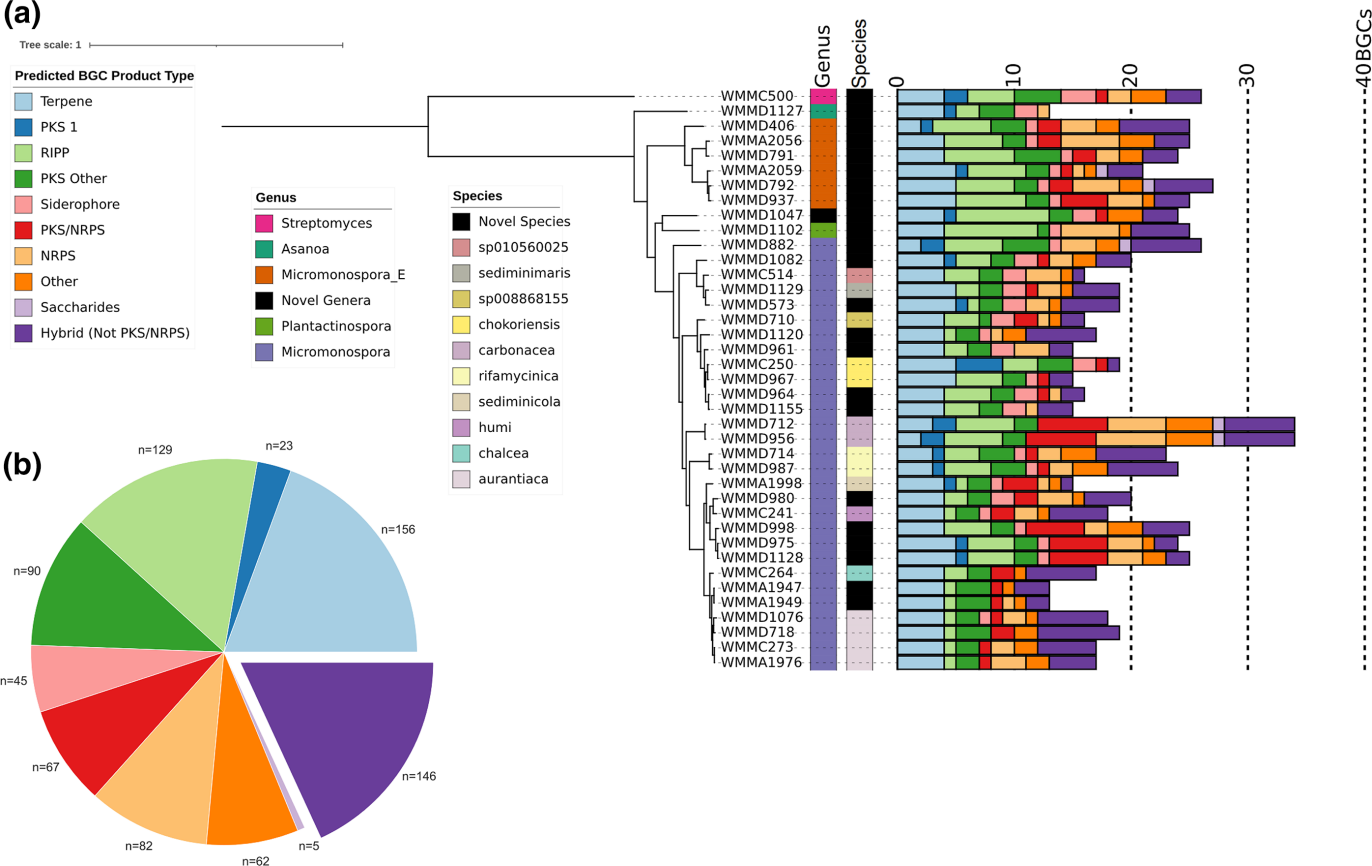
Overall representation of phylogeny and BGCs of *Micromonosporaceae* type strains included in this work. (a) Phylogenetic tree of 38 *Micromonosporaceae* strains and one *Streptomycetaceae* strain (WMMC500). The bar plot represents the antiSMASH v5.1.1 identified BGCs, and colours represent the predicted BGC product type. Bar, 1 substitution per position. (b) Pie chart describing the 779 BGCs identified in the 38 *Micromonosporaceae* strains according to antiSMASH v5.1.1’s predicted product type.

We identified 922 single-copy core orthologues between the genomes of 39 isolates using OrthoFinder v2.5.4 [48]. Multiple sequence alignment of protein sequences was performed for each orthologue using muscle v5 [49] and filtered for sites which featured gaps in more than 10 % of samples using trimAI v1.4 [50]. Filtered protein alignments were concatenated to produce an alignment of length 292 788 aa with coordinates for individual alignments of the 922 orthologues used to generate input for IQ-Tree v2.2.0.3 [51] phylogeny reconstruction using an edge-proportional partition model with ModelFinder Plus [52] and visualized using iTOL v6.6 [53] (Fig. 1a). CompareM v0.1.2 (https://github.com/dparks1134/CompareM) was used to assess average amino acid identity (AAI) between the 39 genomes. The Genome Taxonomy Database (GTDB-tk v2) [30] was used to classify taxonomies for the 39 genomes with GTDB release R207 [54], and the taxonomic identification results are listed in Data S6. FastANI [55] was used to group species across the 39 genomes using ANI, and the species grouping results are listed in Data S6.

### Analysis of intra-BGC diversity using marine *Micromonosporaceae*

**Fig. 2.**
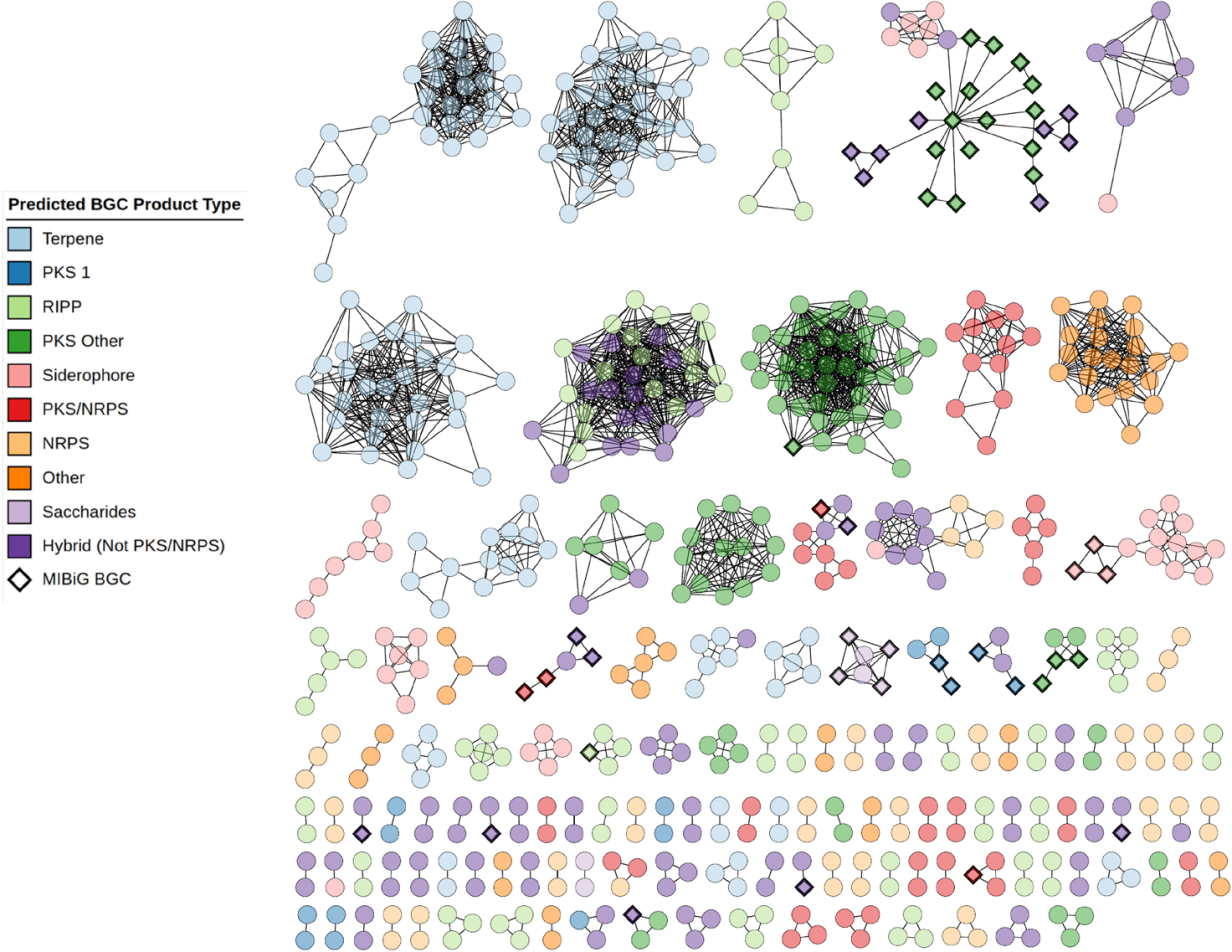
Sequence similarity network (SSN) produced by BiG-SCAPE when analysing *Micromonosporaceae* strains, visualized and annotated with CytoSCAPE. Nodes represent individual BGCs. BGC types are coloured according to the colour key. *Micromonosporaceae* BGCs are represented as circles and MIBiG BGCs are represented as diamonds. Singletons (196 BGCs) have been removed from the visualization, and are shown in Fig. S181.

Utilizing the *Micromonosporaceae* antiSMASH v5.1.1 GenBank files, pairwise distance across predicted BGCs and SSNs was constructed using BiG-SCAPE tool v1.1.0 [34]. This run of BiG-SCAPE was performed at a default cutoff of 0.3 with additional features such as: including BGCs from the MIBiG database v2.1 [56], constructing an all-vs-all distance network mixing all BGC product classes, including BGCs that do not have a distance lower than the cutoff distance specified, and including BGCs with hybrid predicted products into each subclass network. The full parameters for the BiG-SCAPE analysis are listed in Data S8. The network output of BiG-SCAPE was imported into Cytoscape v.3.9.1 [57] (Fig. 2). The BGC product annotations generated by antiSMASH v5.1.1 were categorized based on BiG-SCAPE cluster classes and were added to the Cytoscape visualization. The MIBiG BGC product types were identified using minimal runs of antiSMASH v5.1.1 through ARTS [44, 45], manually annotated using BiG-SCAPE cluster classes categories, and added as annotations to the Cytoscape network.

### Analysis of BGC diversity against terrestrial bacteria

**Fig. 3.**
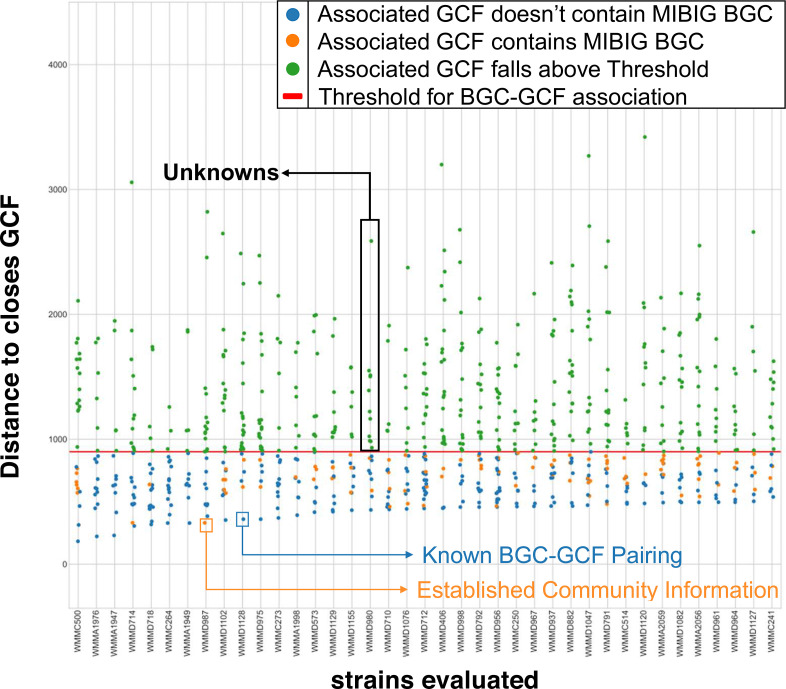
Scatter plot of *Micromonosporaceae* and *Streptomycetaceae* BGCs queried against BiG-SLiCE (threshold=900). Nodes represent individual BGCs. The horizontal red line indicates the threshold for successful clustering of a BGC into a GCF from BiG-SLiCE. The dots are coloured: (a) orange, if the GCF most similar to that BGC contained a BGC from MIBiG; (b) blue, if the GCF most similar to that BGC did not contain a BGC from MIBiG; and (3) green, if the BGC’s distance to the closest GCF fell above the clustering threshold of 900.

*Micromonosporaceae* antiSMASH v5.1.1 outputs were queried against the pre-processed dataset of 29 955 gene cluster family (GCF) models in BiG-SLiCE v1.1.1 [35] using a clustering threshold value of 900. The 29 955 GCF models were previously computed by Kautsar *et al*. [35] using a clustering threshold distance of 900 to group 1 225 071 BGCs into GCFs. For each BGC–GCF pairing, a membership score (distance) was generated, resulting in 779 BGCs having an associated 29 955 distances for each possible GCF. These distances corresponding to BGC–GCF instances were ranked from lowest to highest to generate the top-X hits, where X represents the X-best GCF hits for each BGC, with a lower distance indicating higher confidence in similarity. The full parameters for the BiG-SLiCE runs are listed in Data S8. The file-based SQL database was accessed in Python using the SQLite3 library. The best-performing GCF for each *Micromonosporaceae* BGC was investigated for the presence of MIBiG BGCs and the distance values associated with the BGCs and their topmost GCF pair was plotted in Fig. 3.

### Proposed methodology for identification of novel BGCs

The table of membership scores (distances) between 779 *Micromonosporaceae* BGCs and the 29 955 GCFs in BiG-SLiCE was transformed into a pairwise distance matrix using the Chebyshev distance metric, resulting in a 779×779 matrix describing the distance between BGCs in relation to the distribution of membership scores (distances) across the GCFs. Metric multidimensional scaling (MDS) was performed as a dimensionality reduction technique to reduce the 779×779 matrix into a two-dimensional space [58]. The metric MDS parameters are described in Data S8, and the corresponding metric MDS plots are visualized in Figs 4 and S1–S10. The MDS scatter plot marker size and hue were scaled based on the average distance of the BGC to the top three GCFs (Figs 5 and S11–S20).

**Fig. 4.**
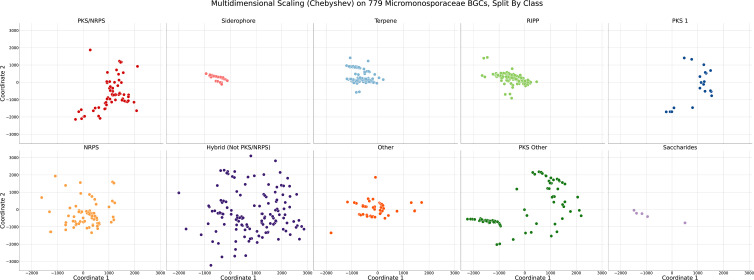
Scatter plots of *Micromonosporaceae* BGCs analysed via multidimensional scaling using Chebyshev pairwise distance. Each dot represents an individual BGC. Distance between BGCs is associated with GCFs in BiG-SLiCE to which they were most similar. Visualization was separated according to predicted BGC product type. The colour code represents the predicted BGC product type.

**Fig. 5.**
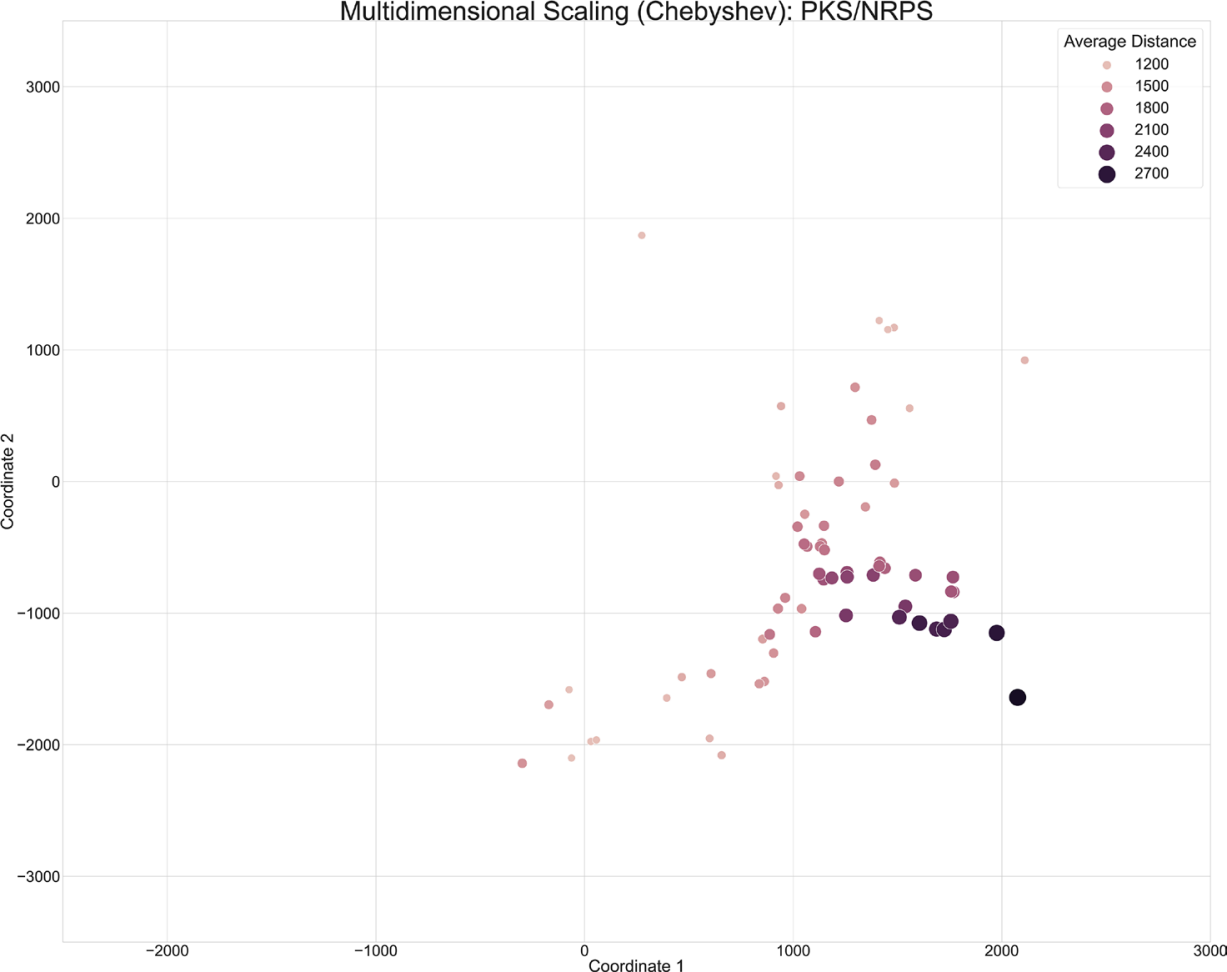
Annotated version of the scatter plot of PKS/NRPS *Micromonosporaceae* BGCs from Fig. 4. The hue and size of the dots were scaled based on the average distance of the BGCs, across the entire dataset, to the top three GCFs in BiG-SLiCE (T=900).

The BGC-GCF table of 779 BGCs and their membership scores (distances) to the 29 955 GCFs in BiG-SLiCE was split into sub-datasets. These sub-datasets were based on the antiSMASH predicted product type of the BGC, resulting in 10 sub-datasets. Each sub-dataset was transformed into pairwise distance matrices, using eight different distance metrics. These metrics were Euclidean, Cosine, Cityblock, Chebyshev, L2, Braycurtis, Canberra and Correlation pairwise distances. The pairwise distance matrices underwent metric MDS to compare our BGCs against each other (Figs 6 and S21–S100). The size and hue of the markers for each BGC in the metric MDS visualizations were scaled based on the average distance of the BGC to the top three GCFs (Figs 7 and S101–S180). The metric MDS parameters are described in Data S8.

**Fig. 6.**
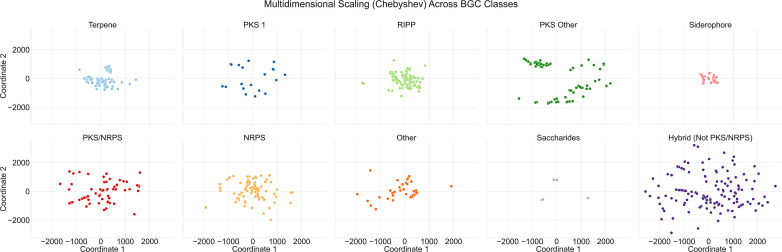
Improved scatter plots of *Micromonosporaceae* BGCs, separated by predicted BGC product type prior to analysis via multidimensional scaling with Chebyshev pairwise distance. Distances between BGCs of a specific product type were associated with most similar GCFs in BiG-SLiCE. Predicted BGC product types are colour coded.

**Fig. 7.**
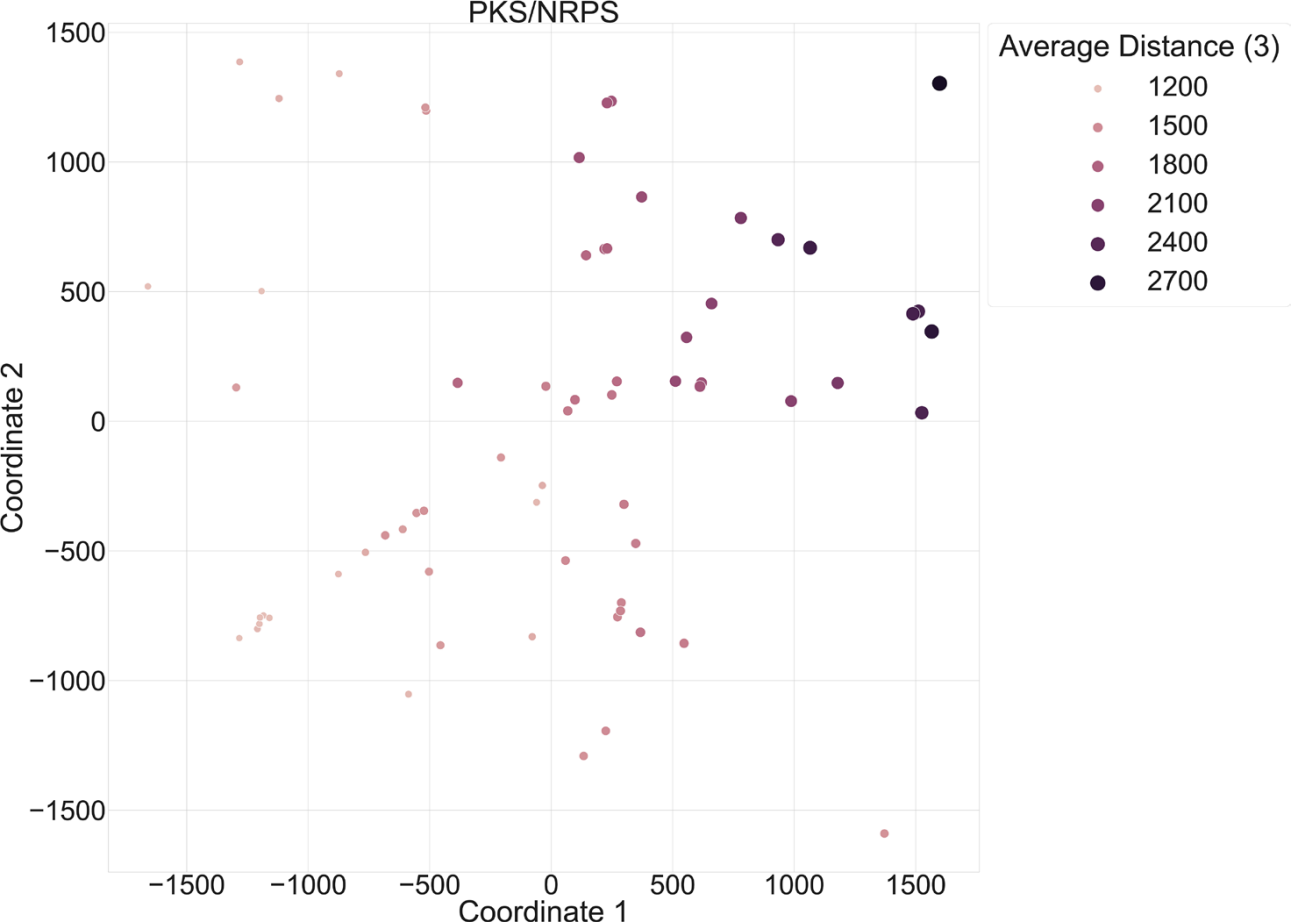
Annotated version of the improved scatter plot of PKS/NRPS *Micromonosporaceae* BGCs from Fig. 6. The hue and size of the dots were scaled based on the average distance of the BGCs, across the predicted BGC product type PKS/NRPS, to the top-3 GCFs in BiG-SLiCE (T=900).

### Statistical analysis of BGC similarity distribution across genera

**Fig. 8.**
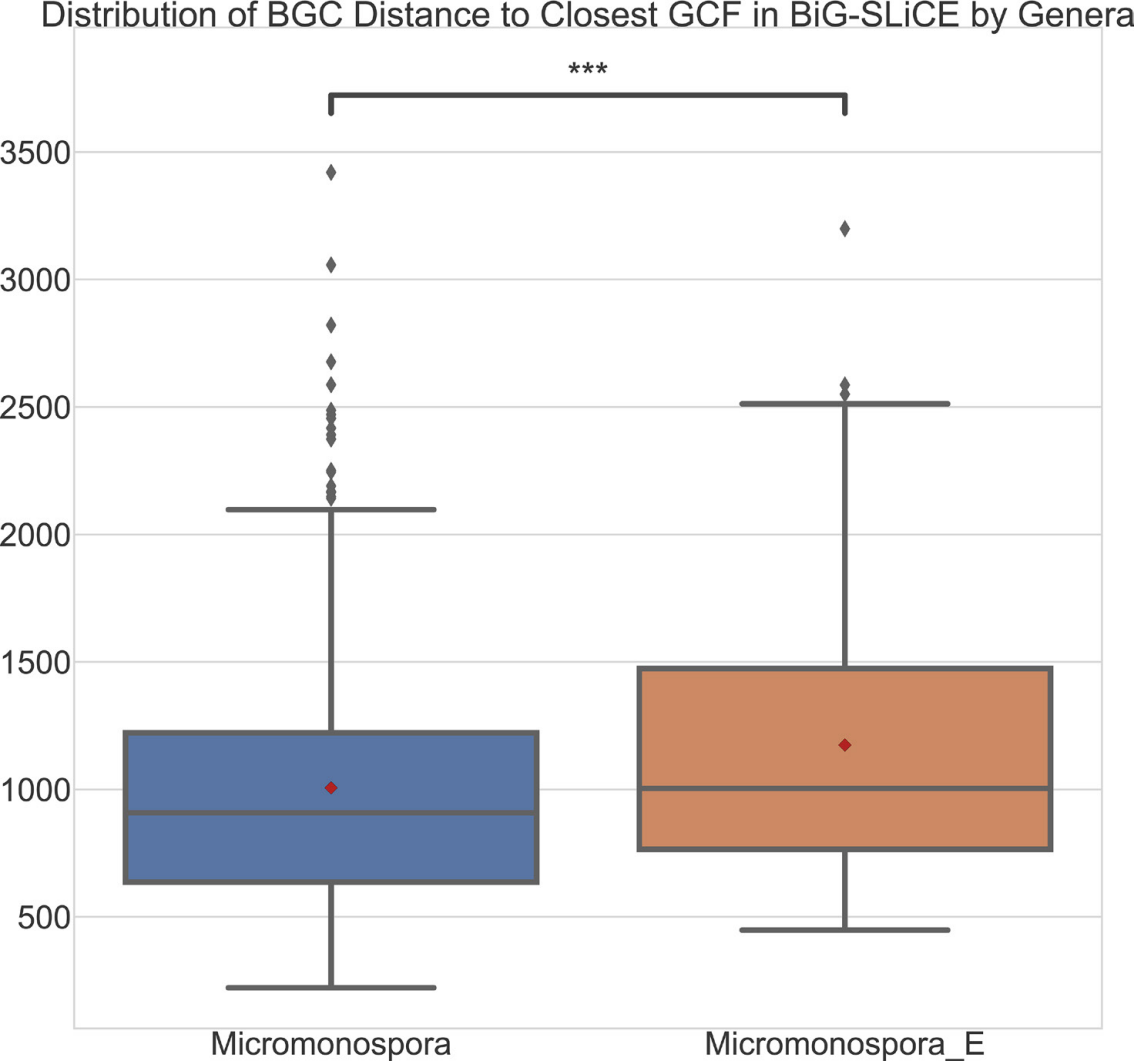
Box and whisper plots comparing the distribution of distances between individual BGCs and the associated closest GCFs in BiG-SLiCE. The distributions have been separated by genera, with six *Micromonospora_E* strains and 29 *Micromonospora*. The red dot represents the means of the distributions. The Mann–Whitney U-test (double-sided) was used to confirm differences in the distributions.

The 570 *Micromonospora* BGCs and 147 Micromonospora_E BGCs were queried against BiG-SLiCE for the best GCF membership score. A comparison of the distributions of BGCs across genera was constructed using a box plot (Fig. 8; Table S1). The Mann–Whitney U-test was applied to evaluate the statistically significant differences across the genera-specific distributions (Table S1). All analyses utilized a significance level of *P*<0.05.

## Results and discussion

Novel NPs from primarily terrestrial bacteria have served as the first and last lines of defence against antibiotic-resistant pathogens for over 90 years [2]. However, trend analyses regarding decades of NP research indicate that terrestrial NP repositories are reaching critical exhaustion levels; in particular, the likelihood of discovering bioactive NPs with clinically employable novel mechanisms of action is becoming diminishingly small. Accordingly, drug discovery initiatives of the last 5–10 years are now aggressively pursuing alternative sources of microbially derived NPs with clinical potential. Inspired by our own interest in marine-derived microbes and the increasingly dire rise of antibiotic resistance, we evaluated the family *Micromonosporaceae*, specifically marine-associated *Micromonosporaceae*, as a promising source of NPs with bioactivity against clinically relevant pathogens. The approach taken, one that critically evaluates BGC content of assorted pools of microbes, enabled us to determine if marine-associated bacteria are sufficiently distinct from previously characterized terrestrial bacteria to warrant in depth marine-focused metabolomics. In this work, we incorporated genomics-based analytics to uncover potential bioactive secondary metabolites unrelated to known molecules based on comparative analyses of microbial BGC content. However, while the dataset used for this study contains a significant number of *Micromonosporaceae* sequenced by our research group, it was not explicitly designed to be a diverse set of bacteria based on phylogenetic grounds and does not represent the entire marine-associated family with regard to metabolic potential.

### Marine bacterial strain genome characteristics

The genome sequences of the 38 selected *Micromonosporaceae* strains, one selected *Streptomycetaceae* strain, associated QUAST (v5.0.2) annotations and their respective BUSCO information are presented in Data S1. The G+C content of the *Micromonosporaceae* strains varied from approximately 70.41 to 73.94 %. Genome sizes ranged from 6 311 079 to 8 920 919 bp, completeness scores ranged from 98.3 to 100 %, and the number of genes ranged from 5688 to 8739.

### Phylogenetic classification of marine bacterial strains

The phylogenetic classifications of the 38 selected *Micromonosporaceae* strains and one *Streptomycetaceae* strain were performed using GTDB-tk v2 (Data S6). AAI and ANI analyses were performed to identify shared genera and shared species, with a minimum threshold of 67.66 % AAI being necessary to belong to the same genera, and an upper bound of 95 % ANI to be considered a novel species strain (Data S6). WMMC500 (*Streptomycetaceae Streptomyces*) was found to be a novel species of *Streptomyces*. WMMD1047 (*Micromonosporaceae*) was uncovered as a novel *Micromonosporaceae* sp. through GTDB-tk v2 taxonomic classification. Utilizing GTDB-tk v2 in conjunction with ANI analysis revealed 18 bacterial strains as belonging to known species of *Micromonosporaceae Micromonospora*. Continued analysis identified 11 bacterial strains as eight novel species of *Micromonosporaceae Micromonospora*. Further investigation discovered six bacterial strains belonging to the genus *Micromonospora_E*, with four novel species of *Micromonosporaceae Micromonospora_E* identified. The designation *Micromonospora_E* comes from GTDB-tk2 splitting the current genus of *Micromonospora* into *Micromonospora*, *Micromonospora_E*, *Micromonospora_G* and *Micromonospora_H* based on AAI [54]. GTDB-tk2 also revealed the presence of two additional novel species, WMMD1127 (*Micromonosporaceae Asanoa*, unknown sp.) and WMMD1102 (*Micromonosporaceae Plantactinospora*, unknown sp.).

Phylogenetic analysis of our 38 selected *Micromonosporaceae* strains and one *Streptomycetaceae* strain broadly conformed to expectation. WMMC500 (*Streptomycetaceae Streptomyces*, unknown sp.) correctly self-identified as a unique clade in our phylogenetic tree and was predicted as a new species of *Streptomyces* that is distinct from the genomes in GTDB-tk2. This result was expected, as *Streptomycetaceae* should clearly diverge from *Micromonosporaceae* through taxonomic classification due to inter-genera differences. However, the discovery of four novel species of *Micromonosporaceae Micromonospora_E* across six bacterial strains emphasizes the diversity of our marine collection, despite being so small. Currently, GTDB release 207 indicates that there are 331 *Micromonosporaceae Micromonospora* genomes, with two corresponding *Micromonosporaceae Micromonospora_E* genomes. Purely from a taxonomic perspective, we have uncovered an additional six *Micromonospora_E* strains that belong to a poorly represented genus in publicly available literature. Additionally, we have also discovered a total of 15 novel species of *Micromonosporaceae*. Even though this dataset was primarily cultivated for *Micromonosporaceae* strains, the taxonomic diversity acquired from collections carried out in only one marine environment hints at a dramatic and unexplored diversity in microbial NPs, clearly warranting future study.

### Diversity and distribution of BGCs in marine *Micromonosporaceae*

The prediction, annotation and characterization of BGCs related to secondary metabolism was generated using antiSMASH v5.1.1. From the 38 *Micromonosporaceae* strains, 779 BGCs were annotated with a distribution of 13–34 BGCs per genome (Fig. 1). Genomes WMMA1949 (*n*=13), WMMA1947 (*n*=13) and WMMD1127 (*n*=13) all had the lowest number of BGCs identified, while WMMD712 (*n*=34) and WMMD956 (*n*=34) carried the highest number of BGCs identified. Manual annotation using antiSMASH-predicted BGC product types using a modified BiG-SCAPE categorization scheme to decipher non-PKS/NRPS hybrid clusters revealed a significant number of hybrid products. Across the Micromonosporaceae strains, the most heavily represented type of BGC was predicted to be involved in the production of terpenes (*n*=156), followed by non-PKS/NRPS hybrids (non-polyketide synthase and non-ribosomal peptide synthetase hybrids) (*n*=146) and RiPPs (ribosomally synthesized and post-translationally modified peptides) (*n*=129).

Terpenes, as the most heavily represented BGC type in our marine *Micromonosporaceae*, represent a structurally and functionally diverse family of natural products. Generally speaking, terpenes share a backbone chain assembly pathway that employs C5 units such as isopentenyl diphosphate (IPP) and dimethylallyl diphosphate (DMAPP) as critical building blocks [7]. Terpenes displaying moderate antibacterial activity against methicillin-resistant *Staphylococcus aureus* (MRSA) and bacteriostatic properties are well established [7]. Terpenoids also have shown antimicrobial, antifungal and anticancer cytotoxic activity [59–61]. Uncharacterized terpenes identified in our BiG-SCAPE analysis represent a largely underexploited NP grouping for possible applications against antimicrobial resistance mechanisms.

Non-PKS/NRPS hybrids were found to comprise the second largest group of BGCs in our *Micromonosporaceae* collection. We defined non-PKS/NRPS hybrids as antiSMASH annotated regions obeying two specific rules: (a) the region contains a hybrid of two or more products; and (b) if the hybrid contains only two products, those products must not only be a Type 1 PKS but also an NRPS. As an example, a non-PKS/NRPS hybrid could have annotations for a Type 2 PKS and RiPP or be composed of a Type 1 PKS, NRPS and terpene. This super-category was constructed to identify potential chemical hybrids that are traditionally underexplored for interesting chemistry. For instance, in WMMC264, an interleaving candidate cluster containing an NRPS, oligosaccharide and terpene overlapping was observed. NPs generally display much greater structural diversity than strictly synthetic agents and accordingly allow for much more extensive probing and utilization of chemical space; this logic is especially spurred on by the abundance of stereochemical features found in vast numbers of NPs [6]. These realizations make clear that further characterization of novel chemical spaces, as illuminated by our NP discovery efforts here, might well broaden synthetic drug approaches for target diversity [6].

RiPPs make up the third largest proportion of BGCs in our collection of *Micromonosporaceae* strains. RiPPs are primarily notable for being structurally diverse and displaying a wide array of biological activities [62], including potent antibiotic [63]/antifungal [64, 65]/anticancer [66, 67] activities, and expressing unique mechanisms of action compared to clinically used drugs. RiPPs are also amenable to biosynthetic engineering to mitigate systemic issues such as poor solubility and limited bioavailability [63]. Of our 779 *Micromonosporaceae* BGCs analysed with antiSMASH v5.1.1, 170 were found to encode a RiPP of some kind. Notably, bacteriocin (*n*=67), lanthipeptide (*n*=58), linear azol(in)econtaining peptides (*n*=37), thiopeptide (*n*=36) and Tfua-related (*n*=20) species comprised over 97 % of the RiPPs identified through antiSMASH (Data S13). Further characterization of these BGCs may reveal particularly malleable RiPPs with good antibiotic activities for engineering against resistant pathogens [63].

Analysis of the predicted BGCs using the BiG-SCAPE workflow revealed 328 GCFs and singletons (Fig. 2). Our analysis identified the 51 MIBiG reference BGCs as similar to our *Micromonosporaceae* BGCs. Specifically, BGCs known to encode the biosynthesis of gentamicin, sisomicin, rosamicin and more were linked to BGCs in our collection (Data S13). Of our 779 *Micromonosporaceae* BGCs, 196 BGCs (~25.1 %) clustered separately into singletons, representing a degree of uniqueness worth studying given their likelihood as beacons of unique chemical chemistry/NPs. The remaining 583 *Micromonosporaceae* BGCs clustered into 132 GCFs seen in Fig. 2, with 83 of the *Micromonosporaceae* BGCs clustering into 16 GCFs containing BGCs from MIBiG, indicating potentially known chemical compound space. The remaining 500 *Micromonosporaceae* BGCs clustered into 116 unique GCFs, traditionally representing unique chemical classes and indicating additional novel chemical entities potentially able to occupy unique chemical spaces. Overall, if all BGCs were sufficiently expressed in lab conditions, we would expect to have observed 312 unique chemical classes from our marine-derived *Micromonosporaceae* collection.

### BiG-SLiCE queries reveal significant likelihood for previously unknown biosynthetic potential

The previous analysis only explored the potential chemical diversity within our *Micromonosporaceae* BGCs. To investigate our marine *Micromonosporaceae* strains’ diversity relative to published terrestrial bacteria sources, BiG-SLiCE was used as a comparative tool. BiG-SLiCE contains over ~1.2 million BGCs from publicly available literature sources, including MIBiG, which have historically been of terrestrial origin due to exhaustive mining of local ecological niches [1]. Specifically, we utilized the BiG-SLiCE model constructed using a clustering threshold value of 900, resulting in 29 955 GCFs against ~1.2 million BGCs, and queried our 779 marine *Micromonosporaceae* BGCs to determine individual BGC similarity to BiG-SLiCE’s GCFs (Fig. 3).

Our analysis revealed 413 BGCs that failed to cluster into the 29 955 GCF model in BiG-SLiCE (clustering threshold T=900); these BGCs thus represent distinctly novel BGCs relative to published literature, an important finding that is perhaps not that surprising given the historical bias towards terrestrial microbes reported in the literature. The remaining 366 marine-derived BGCs that successfully clustered were further categorized, informed by whether or not the closest GCF reported contains a BGC from MIBiG. From this analysis, we uncovered 94 MIBiG-associated *Micromonosporaceae* BGCs. Relatedly, 272 BGCs were identified as being distinct from MIBiG-associated GCFs within BiG-SLiCE. The 94 *Micromonosporaceae* BGCs (MIBiG-associated) probably encode known end products based on their similarity to community annotated BGCs. The remaining 272 BGCs that were not identified as MIBiG-associated may produce known end products but lack meaningful community information that might validate the exact products. In sum, the 413 BGCs that failed to cluster into the BiG-SLiCE model (T=900) represent completely unknown chemical classes, with little to no information regarding potential products beyond antiSMASH annotations. Accordingly, these BGCs showcase the probably vast biosynthetic potential of the marine environment and the microbial diversity it houses.

To contextualize our findings, we combined our BiG-SCAPE analysis and our BiG-SLiCE results to identify novel *Micromonosporaceae* strains that contained novel BGCs in relation to both our own dataset and to published literature. Notably, investigation of WMMD406 (*Micromonosporaceae Micromonospora_E* unknown sp.) in BiG-SCAPE revealed 17 BGC singletons out of the 25 total BGCs identified through antiSMASH. Comparison with BiG-SLiCE queries revealed 21 BGCs that failed to cluster (T=900) into the 29 955 GCFs that represent primarily terrestrial public literature. Of the 17 BGC singletons identified in BiG-SCAPE, 16 were shared across the 21 novel BGCs determined through BiG-SLiCE, indicating potentially unique chemical classes across our dataset and against published literature. Systematic incorporation of BiG-SCAPE analysis and BiG-SLiCE results revealed that the 413 novel BGCs identified through BiG-SLiCE made up 148 of the 196 singletons and 108 of the 132 GCFs visualized in BiG-SCAPE, representing prospective chemical classes (Data S13). Further exploration of marine *Micromonosporaceae* strains could continue to reveal unexplored chemical diversities and their corresponding antimicrobial activities and mechanisms.

### Truly novel BGCs are identifiable using MDS and pairwise distance modelling

Our previous analyses highlighted the merits of combining BiG-SCAPE and BiG-SLiCE to identify strains and BGCs that are distinct within our dataset and also relative to published literature sources. To do this in a systematic fashion, we constructed a pairwise distance matrix (Chebyshev) of our 779 marine *Micromonosporaceae* BGCs using relational information regarding individual BGC distances to all 29 955 GCFs in BiG-SLiCE (T=900). Using metric MDS, we visualized similarity of our marine *Micromonosporaceae* BGCs against each other, split into subplots to show BGC similarity across individual BGC product types (Fig. 4). From these subplots, we observed siderophores, terpenes and RiPPs as primarily co-localizing to similar spaces based on similar GCF distance values in BiG-SLiCE. Notably, the BiG-SLiCE authors have highlighted that BGCs primarily coding for terpenes and RiPPs tend to disproportionately over-cluster due to the presence of fewer extracted features; our observations here are consistent with these earlier postulates [35]. It also is worth noting that, in the context of Fig. 4, the Hybrid (Not PKS/NRPS) category served as the positive control since it is not limited to a singular product class. Variation in this specific case was defined as the largest spread in absolute distance between BGCs defined as belonging to the same category. Manual investigation of Hybrid BGCs revealed that, in specific overlapping cases, an individual component of the Hybrid BGC was shared with the overlapping BGCs in other categories; the instances of overlap were confirmed via blast alignments. Specifically, WMMA1947 region 12, encoding putative siderophore and lanthipeptide units, showed 80.74 % identity in a blast nucleotide comparison with the siderophore subunit’s core biosynthetic gene relative to a siderophore moiety predicted to be produced by WMMD1120 region 14, also a siderophore core biosynthetic gene, seen in Data S12.

To determine how BGCs were localized following metric MDS, we scaled the marker size and hue based on the average distance of the BGC to the top three GCFs in BiG-SLiCE (Fig. 5). Further investigation into specific BGCs that were close in distance revealed that our metric MDS methodology primarily captured variation across BGC product types, but was insufficient to resolve similarity across BGCs belonging to the same predicted BGC product type. Although there is utility in comparing across BGC product types, especially when comparing BGCs that fall within the Hybrid (Not PKS/NRPS) category to other BGC predicted product categories that may share singular components to the Hybrid section, further refinements were clearly warranted.

As such, we separated our BGCxGCF dataset into sub-datasets based on the 10 predicted product types used within this paper, constructed pairwise distance matrices using the distance metrics described earlier, and performed metric MDS to enhance the power of our visualizations to resolve similar BGCs within our *Micromonosporaceae* (Fig. 6).

Utilizing this methodology, manual comparison with BiG-SCAPE’s SSNs and examination of antiSMASH outputs revealed that the distance between BGCs in the metric MDS visualizations corresponded strongly to the presence of shared BGC elements. For instance, WMMD975 region 20, reported in BiG-SCAPE as part of a triplet with WMMD1128 region 24 and D998 region 3, all shared a PKS/NRPS unit. Investigation of the PKS/NRPS metric MDS revealed that WMMD975 region 20 and WMMD1128 overlapped significantly, with WMMD998 region 3 distanced further away. AntiSMASH analysis (v5.1.1) revealed that WMMD975 region 20 and WMMD1128 shared the same core biosynthetic genes for the PKS and NRPS subunits, while WMMD998 contained an additional NRPS core biosynthetic gene, potentially resulting in further modifications to the prospective compound. This methodology allowed us to visually ascertain similarities across BGCs of the same predicted product type, while retaining antiSMASH-level information for the differentiation of BGC pockets.

In particular, scaling the marker size and hue based on the average distance of individual BGCs to their top three GCFs in BiG-SLiCE provided a more visually informative way to identify BGCs deviating from terrestrially derived bacteria (Fig. 7). Analysis of the PKS/NRPS category following Chebyshev-based MDS analysis revealed pockets of BGCs able to serve as representative markers of unique groups. For instance, the information regarding WMMD975’s, WMMD1128’s and WMMD998’s PKS/NRPS subunits were consistent; the average distance to the closest three GCFs in BIG-SLiCE for all BGCs exceeded 2400, indicating the likelihood of novel chemistry. Incorporation of the average distance in our methodology allowed for a visually informative way to further prioritize BGCs that are probably uncharacterized and novel compared to publicly available terrestrial data from BIG-SLiCE’s ~1.2 million BGCs (29 955 GCFs, T=900).

### BGC distribution of *Micromonospora E* is statistically different from *Micromonospora*

*Micromonospora_E* was found to represent an underexploited region for BGCs with potentially novel chemistry. In fact, analysis of the distribution of BGC distances to the closest GCF in BiG-SLiCE uncovered a statistically significant difference between the *Micromonospora* and *Micromonospora_E* BGCs with respect to our data collection. Specifically, the mean BGC distance to the closest GCF in BiG-SLiCE for *Micromonospora_E* was ~1174, compared to *Micromonospora*’s ~1006. Similarly, the median BGC distance to the closest GCF in BiG-SLiCE showed the same trend, with *Micromonospora_E* reporting a median of ~1004, and *Micromonospora* ~908. The Mann–Whitney U-test with a two-sided hypothesis reported a *P*-value of 6.84e-04, less than the significance value of 0.05, indicating that the BGC distance distributions for *Micromonospora* and *Micromonospora_E* are different. In addition, analysis of the number of BGCs reporting a distance to the closest GCF in BiG-SLiCE above the threshold value (T=900), averaged over the number of strains per genera, revealed for *Micromonospora* a total of ~10.03 BGCs er per strain, compared to *Micromonospora_E* with ~14.83 BGCs per strain. Although the BiG-SLiCE pre-processed dataset was developed in 2021, access to future datasets incorporating more publicly available genomes and metagenomes will decrease the overall distances reported. However, this metric makes clear that, if we were to query a marine-derived *Micromonospora* strain’s BGCs against BiG-SLiCE’s currently available pre-processed dataset, we would expect to see approximately 10 BGCs that would report a distance value of ≥900, representing dissimilarity to BiG-SLiCE’s primarily terrestrial dataset, indicating potentially novel chemistry.

## Conclusions

Technological advances of the last decade have had a profound impact on our ability to glimpse molecular potentials made available through the application of microbial organisms. In particular, our ability to correlate genomics information from microbes to putative small molecule products with medicinal potentials is now staggeringly advanced relative to a decade ago. With these advances has come a more complete understanding that historically employed terrestrial environments and their associated microbes may no longer warrant consideration as the optimal source of NP diversity. We provide here a comparative genomic analysis of marine *Micromonosporaceae* with the intention of identifying truly novel BGCs and showcasing that marine-derived organisms now represent an optimal NP source when it comes to considerations of structural and functional NP diversity. Our analyses revealed the biosynthetic diversity of marine *Micromonosporaceae*, especially in comparison to publicly curated databases focused on terrestrially derived genomes. Moreover, focused dissection and comparisons of the 39 individual genomic datasets chosen to showcase our bioinformatics logic allowed us to identify several novel species belonging to *Micromonosporaceae Micromonospora_E*. In particular, this discovery represented a 4-fold increase in known *Micromonosporaceae Micromonospora_E* compared to the 311 480 bacterial strains stored in GTDB-tk2 from publicly available data. Beyond the basic nuts and bolts discoveries relating marine-derived to terrestrially derived microbes and the clearly superior diversity of marine systems relative to terrestrial ones, we also endeavoured to develop a novel methodology to visually explore BGC similarities across (and within) strain collections and against publicly available data. In sum, the computational campaign disclosed here showcases: (a) the utility of prioritizing BGCs based on novelty, relative to large existing databases; and (b) the potential of renewed investigations of marine *Micromonosporaceae* as an outstanding repository for NP scaffolds, based largely on BGC content.

## Repositories

Please note that all genome sequence data employed in this study have been deposited with NCBI. Strains and their corresponding accession numbers are as follows:

**Table:** 

Strain name	Accession (whole genome)	Strain name	Accession (whole genome)
WMMA1363	JAUALB000000000	WMMD980	GCA_029626035.1
WMMB482	GCA_019038635.1	WMMD987	GCA_029581435.1
WMMC250	GCA_027460215.1	WMMD937	GCA_029581175.1
WMMA1976	GCA_027497335.1	WMMD964	GCA_029581455.1
WMMA1998	GCA_027497375.1	WMMD998	GCA_029581235.1
WMMB486	JAUALC000000000	WMMD1129	GCA_029581255.1
WMMA1947	GCA_027497355.1	WMMD1155	GCA_029581275.1
WMMA1949	GCA_027460145.1	WMMD406	GCA_029626025.1
WMMA2056	CP128360	WMMD712	GCA_029581295.1
WMMA2059	GCA_027497315.1	WMMD714	GCA_029581315.1
WMMC241	GCA_027460165.1	WMMD718	GCA_029626075.1
WMMC264	GCA_027497275.1	WMMD792	GCA_029626105.1
WMMC273	GCA_027460285.1	WMMD956	GCA_029626125.1
WMMC514	GCA_027497295.1	WMMD961	GCA_029626145.1
WMMC500	GCA_027497195.1	WMMD967	GCA_029626165.1
WMMD882	GCA_027497255.1	WMMD1047	GCA_029626155.1
WMMD1128	GCA_027497235.1	WMMD1076	GCA_029581475.1
WMMD573	GCA_027497175.1	WMMD1082	GCA_029626175.1
WMMD710	GCA_029626045.1	WMMD1102	GCA_029626265.1
WMMD791	GCA_029581195.1	WMMD1120	GCA_029626235.1
WMMD975	GCA_029581215.1	WMMD1127	GCA_029626225.1
